# Posterior reversible encephalopathy syndrome in severe leptospirosis: A case report

**DOI:** 10.1016/j.idcr.2025.e02330

**Published:** 2025-07-26

**Authors:** Thamalee Palliyaguru, Pramith Ruwanpathirana, Mythily Aravinthan, Dilshan Priyankara, Praveen Weeratunga

**Affiliations:** a Professorial unit in medicine, National Hospital of Sri Lanka, Colombo, Sri Lanka; b Department of Clinical Medicine, Faculty of Medicine, University of Colombo, Colombo, Sri Lanka; c Flinders Medical Centre, Bedford Park, SA 5042, Australia; d Medical Intensive Care Unit, National Hospital of Sri Lanka, Colombo, Sri Lanka

**Keywords:** Leptospirosis, Posterior reversible encephalopathy syndrome, Acute kidney injury, Infection

## Abstract

**Introduction:**

Posterior reversible encephalopathy syndrome (PRES) is a clinico-radiological entity with diverse aetiologies. It presents with headache, altered sensorium, seizures, and visual disturbances and is characterised by symmetrical white matter changes on neuroimaging. An acute rise in the blood pressure is the commonest cause of PRES. We report a patient who developed PRES in the recovery phase of severe leptospirosis. We discuss the interplay of possible patho-mechanisms of PRES in leptospirosis.

**Case presentation:**

A 14-year-old Sri Lankan male presented with a 5-day history of high-grade fever and myalgia and a 2-day history of oliguria. Physical examination was unremarkable. Leptospirosis was diagnosed using the microscopic agglutination test. During hospitalisation, he developed acute kidney injury and pulmonary haemorrhage, requiring mechanical ventilation and treatment with intravenous ceftriaxone and daily plasma exchange. Both complications resolved by day 13 of the illness. On day 14, he developed sudden-onset altered consciousness followed by a generalised tonic-clonic seizure. There were no signs of meningism, and serum glucose, calcium, magnesium, and sodium levels were within normal limits. Brain MRI demonstrated symmetrical T2/FLAIR hyperintensities in the bilateral parieto-occipital white matter, consistent with PRES. Blood pressure at the time was 140/90 mmHg. Cerebral angiography excluded vasculitis. Neurological symptoms resolved spontaneously within 24 h, and the patient recovered fully.

**Conclusion:**

PRES is an uncommon complication of severe leptospirosis. Clinicians should consider this diagnosis in patients with leptospirosis who develop acute encephalopathy or seizures.

## Introduction

Leptospirosis is a zoonotic infection caused by the spirochete *Leptospira interrogans* [1], [2]. It affects 1 million people annually, with a global case fatality of 5.9 % [3].

Leptospirosis is usually an acute febrile illness that resolves uneventfully. However, a subset of patients develops severe disease characterised by organ involvement, including pulmonary haemorrhage, acute kidney and liver injury, myocarditis, cytopenias, pancreatitis, aseptic meningitis and multiorgan dysfunction [4]. These complications result from direct tissue invasion by the spirochete and immune-mediated injury secondary to an exaggerated host response.

Posterior reversible encephalopathy syndrome (PRES) is a clinico-radiological syndrome with acute onset cerebral symptoms and characteristic radiological features [5], [6]. Its clinical features include headache, altered mental status, seizures, visual disturbances (including cortical blindness), and focal neurological deficits. In magnetic resonance imaging (MRI), bilateral symmetrical T2 and FLAIR white matter hyperintensities are seen without diffusion restriction. These lesions are found predominantly in the parieto-occipital regions. Although less sensitive, CT imaging may reveal corresponding hypodensities in affected areas. The commonest cause of PRES is severe hypertension.

We describe a patient who developed severe leptospirosis complicated with acute kidney injury and pulmonary haemorrhage and developed PRES.

## Case presentation

A 14-year-old Sri Lankan male was admitted with five days of high-grade fever, myalgia and reduced urine output for two days. His past medical and family history was unremarkable. He reported exposure to stagnant water in a paddy field approximately two weeks before presentation.

On examination, his temperature was 38.1° C. The blood pressure was 90/50 mmHg, and the pulse rate was 112 beats per minute. This blood pressure was low for his age and sex, with the systolic and diastolic pressures corresponding to the 1st and 11th percentiles, respectively. His height was 177 cm, corresponding to the 87.5th percentile. The respiratory and neurological examination was unremarkable. There was no icterus, mucosal or conjunctival haemorrhages.

The initial investigations revealed a total leucocyte count of 18 × 103/μl (reference: –4,11), a platelet count of 54 × 103/μl (150 – 450), and a normal haemoglobin. His C reactive protein (CRP) was 232 mg/dl (<6), and the serum creatinine was 2.46 mg/dl (0.4 – 1.1). There was no evidence of liver injury or coagulopathy. Leptospirosis was confirmed with a microscopic agglutination test (MAT) titre of 1:160. Blood cultures and microbiological evaluation for Mycoplasma, COVID-19 and influenza were negative.

He was treated with intravenous (IV) ceftriaxone 1 g 12 hourly. We managed the acute kidney injury conservatively by maintaining the potassium, fluid and acid-base balance. The maximum serum creatinine (4.8 mg/dl) occurred on the eighth day of illness. The patient did not require renal replacement therapy.

On day six of illness, the patient developed acute pulmonary haemorrhage evidenced by declining oxygen saturation, bilateral fluffy shadows in the lung fields (see supplementary material 1 for high resolution computed tomography (HRCT) images) and a dropping haemoglobin level. We intubated and mechanically ventilated the patient for type 1 respiratory failure. We followed the standard protocol of five cycles of daily plasma exchanges (60 mL/kg, using fresh frozen plasma) and intra-nasal desmopressin (20 µg, six hourly) for leptospirosis pulmonary haemorrhage syndrome. We did not treat him with IV corticosteroids.

By the 12th day of illness, the patient was clinically improving with reducing ventilatory support requirement. The serum creatinine approached the baseline, and the urine output improved. We extubated him on the 13th day of illness.

On the 14th day of illness, the patient acutely became confused and agitated. A few hours later, he developed a generalised tonic-clonic convulsion lasting for 2 min, followed by post-ictal drowsiness. We reintubated him to maintain the airway.

**Fig. 1:**
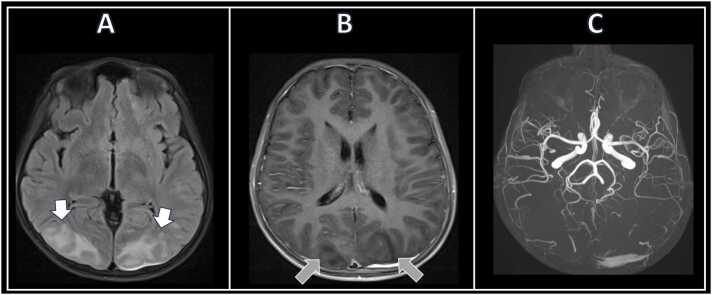
Magnetic resonance imaging of the patient when he developed posterior reversible encephalopathy syndrome. Note the white matter hyperintensities (white arrow) in the FLAIR (subset A) sequence and the corresponding hypo-intensities (grey arrow) in the T1 (subset B) sequence. The subset C depicts the magnetic resonance angiogram. There were no changes of cerebral vasculitis. Only the occipital lobe involvement is shown. FLAIR: Fluid-attenuated inversion recovery.

His blood pressure was 140/90 mmHg, corresponding to the 98th and 99th percentiles for systolic and diastolic pressure, respectively. There was no neck stiffness or papilledema. His serum sodium was 138 mmol/L (135 – 145), and his blood glucose was 148 mg/dl (70 – 180). Calcium and magnesium levels were normal. There were bilateral occipital white matter hypodensities in the non-contrast CT scan of the brain. MRI (magnetic resonance imaging) of the brain revealed bilateral symmetrical T2 signal hyperintensities in the occipito-parietal white matter compatible with PRES (Fig. 1). There were no features of vasculitis in the magnetic resonance angiogram (MRA) or evidence of cerebral venous thrombosis in the magnetic resonance venogram (MRV). Because we made a positive diagnosis of PRES, we did not perform a lumbar puncture.

We reduced the blood pressure to 110/80 mmHg and maintained that with IV labetalol. His consciousness improved over 24 h, after which we extubated him.

**Fig. 2:**
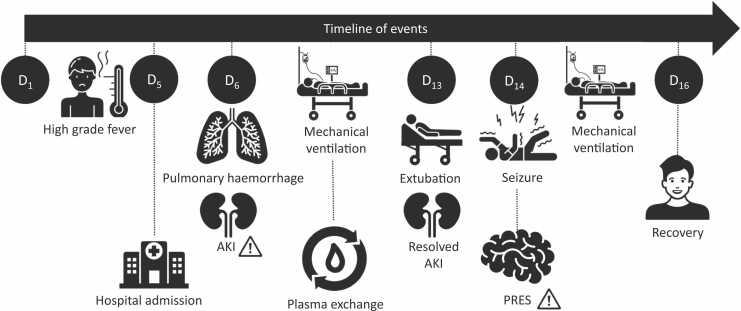
The timeline of events. The days are numbered from the onset of illness. AKI: Acute kidney injury. PRES: Posterior reversible encephalopathy syndrome.

The patient did not develop any further seizures or neurological sequelae. Follow-up neuroimaging was normal at 3 months. The patient had an uneventful recovery. The timeline of events is given in Fig. 2.

## Discussion

**Fig. 3:**
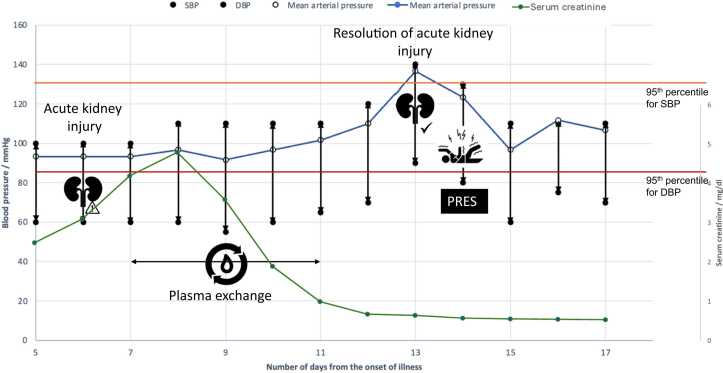
Fluctuation of blood pressure and serum creatinine during the hospital stay. Blood pressure was measured manually 4 times a day using a sphygmomanometer and the average value was rounded to the closest multiple of ten. Note the elevation of blood pressure in the 48 h preceding the seizure. The dark blue line depicts the mean arterial pressure. The 95th centiles for systolic and diastolic blood pressures for a 14-year-old boy are given in orange and red lines, respectively. The green line illustrates the progression of serum creatinine. (right axis). SBP: Systolic blood pressure. DBP: Diastolic blood pressure. MAP: Mean arterial pressure.

We describe a rare occurrence of PRES in the recovery period of severe leptospirosis. In children, blood pressure exceeding the third standard deviation (99.8th percentile) has a 91 % sensitivity and 85 % specificity in predicting PRES [7]. While our patient had a blood pressure in the 98th – 99th percentile range (Fig. 3), attributing PRES solely to elevated blood pressure oversimplifies the underlying pathophysiology

Rather than sustained hypertension alone, PRES is increasingly associated with blood pressure fluctuations and endothelial dysfunction [8]. An acute rise in blood pressure can overwhelm cerebral autoregulation, leading to cerebral hyperperfusion, endothelial injury, and blood-brain barrier disruption, causing vasogenic oedema [9]. Blood vessels in the posterior circulation lack an autonomic innervation [10] and have a lower capacity for autoregulation. Therefore, the parieto-occipital lobes are predisposed to hyperperfusion in acute blood pressure fluctuations [11]. Paradoxically, an acute rise in blood pressure causes endothelial injury, vessel occlusion and hypoperfusion in certain parts of the brain [12], [13]. Hypoperfused areas have cytotoxic oedema (due to cell swelling) instead of vasogenic oedema.

We postulate that blood pressure fluctuations in our patient were driven by dynamic alterations in the renin-angiotensin-aldosterone system and sympathetic tone during AKI recovery.

PRES occur in the absence of acute hypertension and blood pressure fluctuations if there is direct endothelial injury [11]. This is well described with immunomodulatory drugs and renal and autoimmune diseases [11], [14].

Leptospirosis exerts multiple pathogenic effects on endothelial cells. Leptospira disrupts the endothelial glycocalyx [15], adheres, invades [16], and activates [17] the endothelial cells, triggering the secretion of pro-inflammatory cytokines. We propose that primary endothelial injury due to leptospirosis lowers the threshold for PRES development. Infections with endothelial injury, such as gram-positive septicaemia [18], viral infections (HIV[19], COVID-19 [20]), rickettsial infections [21], and malaria are known to cause PRES.

Acute kidney injury (AKI) is an independent risk factor for PRES [22], [23]. It has been reported in postinfectious glomerulonephritis [24], lupus nephritis, AKI secondary to rhabdomyolysis [25] and extensive haemolysis [26]. PRES in AKI likely reflects the effects of endothelial injury (due to metabolic disturbances) and blood pressure fluctuations.

**Fig. 4:**
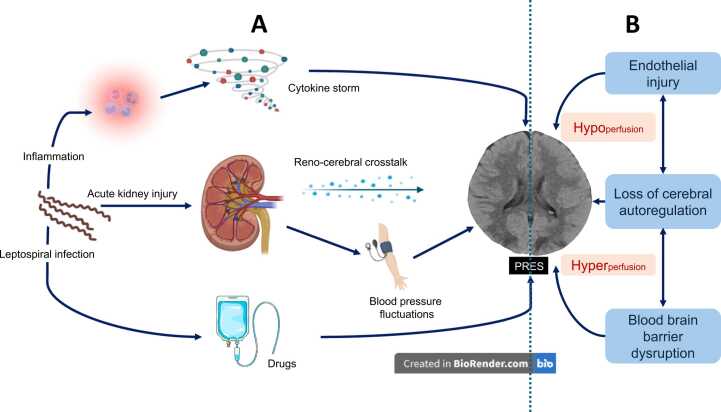
Panel A illustrates the potential pathophysiological mechanisms for posterior reversible encephalopathy syndrome (PRES) in leptospirosis. Leptospirosis induces an acute inflammatory reaction, which can progress to a cytokine storm. The cytokines can lead to endothelial dysfunction and cause a PRES. Leptospirosis causes an acute kidney injury (AKI). Acute kidney injury is a risk factor PRES. The cross-talk between the kidney and the brain possibly modulates PRES in AKI. Furthermore, blood pressure fluctuations in AKI can lead to PRES. Certain drugs used to treat leptospirosis can induce PRES, although this is not relevant to our case. Subset B illustrates the pathogenesis of PRES. Any aetiology that causes PRES does so by causing endothelial injury, disruption of vascular autoregulation and blood-brain barrier disruption. These changes can cause both cerebral hyper and hypo-perfusion. Vasogenic oedema develops in areas of hyper-perfusion, and cytotoxic oedema develops in hypo-perfusion. The non-contrast CT brain in this image is the actual CT of the patient discussed in the case.

Our patient illustrates how multiple mechanisms interplay- resulting in hyper and hypo-perfusion of the brain (Fig. 4). Given the prevalence of these risk factors, PRES in leptospirosis can be expected to be a common complication, but it is not. Reviews and textbooks on leptospirosis do not recognise PRES as a complication.

We identified three reports of leptospirosis complicated with PRES [27], [28], [29] (Table 1). Seizures were the initial presentation in each case, and all patients had AKI and high blood pressure. Each made a full recovery. In addition, Saeed et al. reported a case of leptospirosis presenting with encephalopathy, status epilepticus, and cortical blindness, where the diagnosis of PRES was not considered due to the absence of hypertension [30]. We propose that this, too, could represent PRES.

**Table 1:** Literature review on leptospirosis-associated PRES.

Author			J Aram et al. 2010 UK[27]	Priyankara et al., 2019 Sri Lanka[29]	Lakmali et al., 2021 Sri Lanka[28]	Index case 2024 Sri Lanka
Age (years); gender			34, Male	29; Male	21; Male	14; Male
Organs involved	AKI		√	√	√	√
	ALI		√			
	Pulmonary h’ages			√		√
	Myocarditis			√		
	Rhabdomyolysis				√	
Day of illness when PRES occurred			Day 21	Day 10 Recovery phase	Day 08 Recovery phase	Day 14 Recovery phase
Symptoms/ signs		Confusion	√	√		√
		headache	√		√	
		Seizures	√	√	√	√
		Cortical blindness	√		√	
		BP (mmHg)	170/90	160/120	150/90	140/90
Outcome			Complete recovery	Complete recovery	Complete recovery	Complete recovery

AKI – Acute kidney injury, ALI – Acute liver injury, Pulmonary h’ages – Pulmonary haemorrhages, BP – blood pressure PRES – Posterior reversible encephalopathy syndrome

It is plausible that endogenous protective mechanisms limit the development of PRES despite risk factors. Emerging evidence suggests that genetic polymorphisms affecting endothelial function, cerebral autoregulation, and immune signalling may predispose some individuals to PRES. Additionally, strain-specific differences in Leptospira virulence may influence the extent of endothelial injury. Unfortunately, we were unable to serotype the organism due to resource limitations.

In conclusion, PRES is a rare yet important complication of severe leptospirosis. We hypothesise that its pathogenesis is multifactorial, involving blood pressure variability, acute kidney injury, and inflammatory endothelial injury. PRES should be considered in any patient with leptospirosis presenting with seizures or altered consciousness, particularly during the recovery phase.

## Ethics approval and consent to participate

Approval from an ethics review committee was not sought as the publication is a case report per institutional policy (Ethics Review Committee of the National Hospital of Sri Lanka). We adopted the principles of the Declaration of Helsinki in collecting data and reporting.

## Funding

No funding involved.

## CRediT authorship contribution statement

**Pramith Ruwanpathirana:** Writing – original draft, Conceptualization. **Thamalee Palliyaguru:** Writing – original draft, Conceptualization. **Dilshan Priyankara:** Writing – review & editing, Supervision, Conceptualization. **Mythily Aravinthan:** Investigation, Conceptualization. **Praveen Weeratunga:** Writing – review & editing, Supervision, Conceptualization.

## Declaration of Competing Interest

The authors declare that they have no known competing financial interests or personal relationships that could have appeared to influence the work reported in this paper.

## Data Availability

The patient's clinical records can be obtained from the authors upon reasonable request.

## Footnotes

Appendix A Supplementary data associated with this article can be found in the online version at doi:10.1016/j.idcr.2025.e02330.
