# Supplementary figures and images for: Posterior reversible encephalopathy syndrome in severe leptospirosis: A case report
Source: IDCases. 2025 Jul 26;41:e02330. doi: 10.1016/j.idcr.2025.e02330 (PMC12320078; doi:10.1016/j.idcr.2025.e02330)

## Slide 1
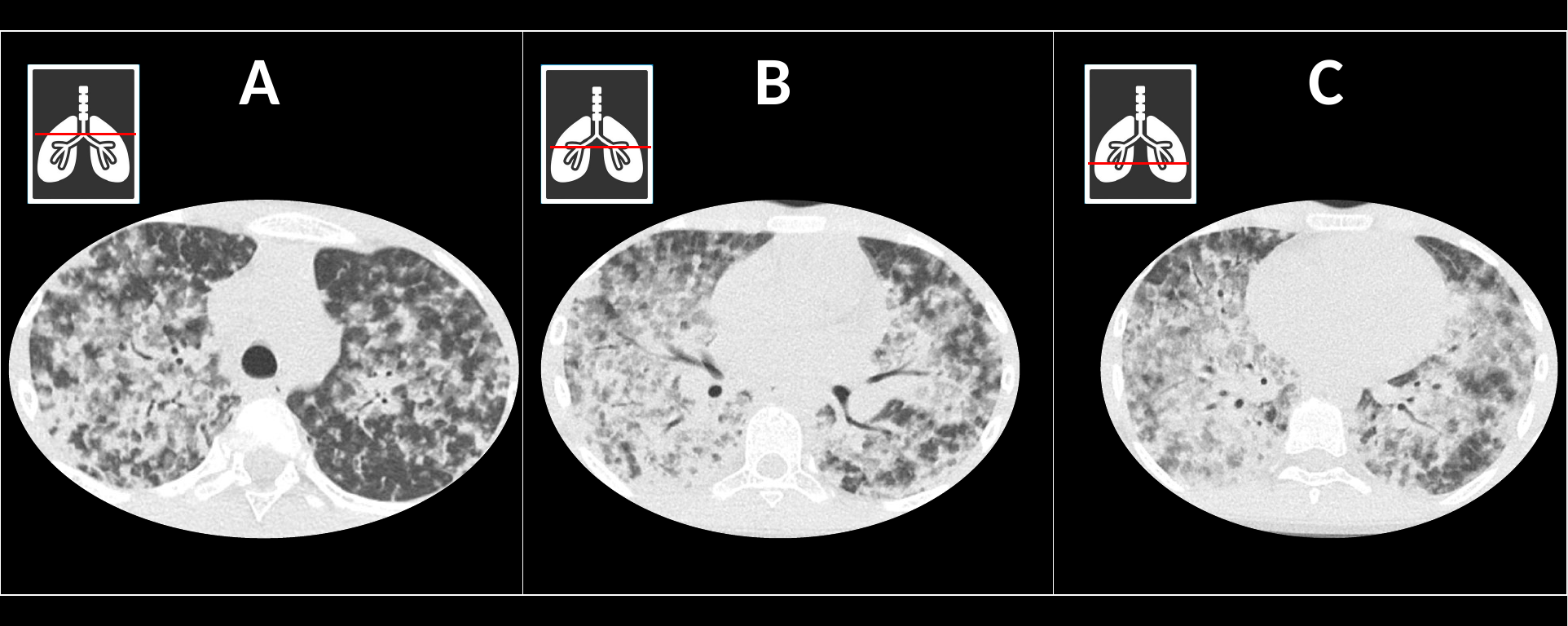

A
B
C

Supplement: Supplementary file 1 — Supplementary material [file mmc1.pptx]
